# Carotenoid-Related Volatile Compounds of Tobacco (*Nicotiana tabacum* L.) Essential Oils

**DOI:** 10.3390/molecules24193446

**Published:** 2019-09-23

**Authors:** Venelina Popova, Tanya Ivanova, Tsvetko Prokopov, Milena Nikolova, Albena Stoyanova, Valtcho D. Zheljazkov

**Affiliations:** 1 Department of Tobacco, Sugar, Vegetable and Essential Oils, University of Food Technologies, 4002 Plovdiv, Bulgaria; vpopova2000@abv.bg (V.P.); tantonieva@mail.bg (T.I.); aastst@abv.bg (A.S.); 2 Department of Engineering Ecology, University of Food Technologies, 4002 Plovdiv, Bulgaria; tsvetko_prokopov@abv.bg (T.P.); milena_nikolova86@abv.bg (M.N.); 3 Department of Crop and Soil Science, 3050 SW Campus Way, Oregon State University, Corvallis, OR 97331, USA

**Keywords:** tobacco, essential oils, volatile compounds, carotenoids

## Abstract

Tobacco (*Nicotiana tabacum* L.) aroma is an important attribute of tobacco quality and is influenced by a variety of minor chemical components, including carotenoid degradation products. The objectives of this work were to determine the content of the most important fragrance-shaping carotenoid degradation products in the essential oils (EOs) of the three types of Bulgarian tobacco—Oriental (OR), flue-cured Virginia (FCV), and Burley (BU)—and to compare them with other aromatic products from tobacco. The content of total carotenoids and β-carotene was highest in BU tobacco (22.23 and 20.34 mg/100 g DW, respectively), followed by OR (13.60 and 12.09 mg/100 g DW in variety “Plovdiv 7” (Pd7); 6.27 and 5.45 mg/100 g DW in “Krumovgrad” (Kr), and FCV (5.93 and 3.73 mg/100 g DW). EOs were obtained by hydrodistillation in an acidified medium, and the main aroma-impact compounds from carotenoid degradation (identified by GC-MS) were as follows: FCV-α-ionone (0.61 mg/100 g DW), dihydro-β-ionone (0.96 mg/100 g DW), β-damascenone (1.26 mg/100 g DW); BU-α-ionone (0.73 mg/100 g DW), dihydro-β-ionone (1.19 mg/100 g DW), β-damascenone (1.35 mg/100 g DW); OR(Kr)-α-ionone (0.20 mg/100 g DW), β-ionone (1.08 mg/100 g DW), dihydro-β-ionone (1.34 mg/100 g DW), β-damascenone (0.36 mg/100 g DW); OR(Pd7)-α-ionone (1.43 mg/100 g DW), dihydro-β-ionone (1.73 mg/100 g DW), β-damascenone (1.23 mg/100 g DW). Ionone and its derivatives were not identified in the aroma extraction products concrete, resinoid, or absolute. The results suggest that temperature, pH of the medium, process duration, and possibly other unknown factors affect carotenoid transformation. The study provides insight into the composition of tobacco EOs and may be of interest to the fragrance industry.

## 1. Introduction

Tobacco (*Nicotiana tabacum* L.) has been a focus of scientific research for centuries due not only to its religious, ethnobotanical, medicinal, physiological, and social role in human history, but also due to its importance as an aromatic plant. It has turned into one of the most extensively studied natural materials, and its chemical composition and diverse beneficial and harmful effects are widely popularized [1,2]. As Mookherjee and Wilson [1] state, “no natural product in the flavor and fragrance industry can match tobacco for the number of volatile constituents which have been identified” and even “what is not in tobacco is not in any other natural product”. More than 3000 volatile and nonvolatile components in tobacco have been listed in scientific reports, representing a great variety of aroma-impact chemicals including nitrogenous compounds, sulfur compounds, terpenoids, carotenoid degradation products, labdanoids, and various aromatics.

There is abundant research devoted to the identification of the aroma-shaping compounds in tobacco leaves (including carotenoids and their degradation products), as well as in tobacco essential oil (EO). In general, this research has mainly targeted evaluation of tobacco quality or opportunities for improving or mimicking tobacco aroma. Considerably fewer reports focus on the obtaining of standard (traditional, established) natural aromatic products from tobacco intended for perfumery and cosmetics [3]. These aromatic products typically include tobacco concrete and tobacco absolute, used in fine perfumery (“tabac”, “dry” or “masculine” scented perfumes), and tobacco resinoid, used in the process of casing or top flavoring of tobacco blends for cigarettes and other tobacco products [4]. They are obtained from cured and aged tobacco leaves, exclusively from the Oriental and cigar types, by extraction with organic (polar or non-polar) solvents, under well-established technologies. Lately, tobacco absolute is obtained by supercritical CO_2_ extraction and is provided for use in perfumery and as a tobacco flavor enhancer. Generally, there are three major types of tobacco (or four—if Maryland is included, depending on the market) that are used as blends in the manufacture of cigarettes: Oriental tobacco (also known as aromatic, Turkish, or Greek tobacco), flue-cured tobacco (Virginia bright), and Burley (light air-cured tobacco). Each of these types displays very specific organoleptic properties, with Oriental tobacco known for having the richest flavor. The three major types of tobacco are all traditionally produced in Bulgaria; all are highly valued in the international market, especially the Oriental type. Plant materials from all three types of tobacco produced in Bulgaria are potential sources of aromatic products. Production of aromatic products is a promising niche, considering current European Union restrictions and reduction in the subsidized production of tobacco intended for direct consumption. Historically, tobacco concrete, resinoid, and absolute have been obtained via extraction from high quality fermented Oriental tobacco in the country since the late 1960s.

Tobacco aroma is an important quality attribute of tobacco and is dependent on a large number of minor chemical constituents [5,6,7,8,9], among which are carotenoid degradation products.

Tobacco is one of the richest sources for degraded carotenoids (also labeled as norisoprenoids, norterpenoids, or nor-carotenoids), with almost 100 chemical constituents being identified [10]. In a comprehensive review on tobacco isoprenoids and their degradation products, including many important aroma components, Wahlberg and Enzell [2] emphasized that although some degraded isoprenoids are generated de novo during the curing of tobacco leaf, many do occur in fresh green leaf. Similar findings were reported earlier by Fujimori et al. [11]. Four major carotenoids are present in green tobacco (lutein, *β*-carotene, violaxanthin, and neoxanthin), along with multiple other minor or trace carotenoids. In green leaf, total carotenoids comprise approx. 2000 mg kg^−1^ and undergo 80–95% decrease in concentration after maturation, senescence, curing, and aging [12,13].

Many investigations have elucidated the mechanisms driving carotenoid transformation to the large number of carotenoid metabolites encountered in tobacco. Most of these studies hypothesized that oxidative degradation, both enzymatic and autoxidative in nature, of carotenoids and other terpenoids led to formation of ionones and ionone-related substances [10]. The transformation of lutein and β-carotene by oxidation [14], thermal degradation [15,16], thermal degradation under reduced pH value [11,17,18,19], specific microbial degradation [20], and other pathways have all been determined to produce the different nor-carotenoid components found in tobacco.

**Figure 1:**
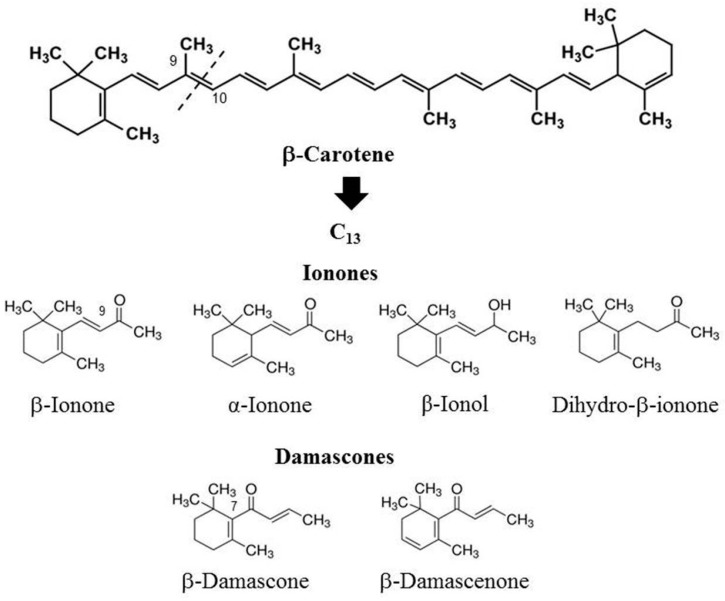
Carotenoid-related compounds in the study.

Oxidative cleavage of the carotenoid chain may occur in a number of locations (bonds from 6–7 to 9–10, respectively) resulting in a variety of compounds from the classes of C_9_ to C_13_-isoprenoids, many of which are important fragrances and constituents of tobacco flavor [10,19]. The C_13_-norisoprenoids with an oxygen function in the side chain include two basic families of constituents, differing by the position of the oxygen function—at carbon atom 9 for the ionones and at carbon atom 7 for the damascones. A simplified scheme of carotenoid-derived compounds related to this study is presented in Figure 1.

In previous studies [21,22] we carried out a GC-MS profiling of the EOs and the traditional aromatic products (i.e., concrete and resinoid) of different Bulgarian tobaccos, but their carotenoid-related composition was not discussed in detail, although data suggested differences on both product and tobacco type basis. Therefore, we hypothesized that an insight into the carotenoid-related composition of tobacco EOs and other tobacco aroma products in a direct comparison between different tobaccos, as well as into the factors involved, would agree with previous findings about carotenoid transformation in other plant materials and in other tobaccos. Furthermore, this will contribute to contemporary natural product investigation. We further hypothesized that the differences in the carotenoid-derived compounds in tobacco EOs and in the traditional extraction aroma products, such as concrete and resinoid, would be related to the specific conditions for obtaining the respective product, and in particular to the effect of thermal degradation and reduced pH during the hydrodistillation of the EOs. The objectives of this work were to provide an insight into the composition of Bulgarian tobacco EOs, on the basis of a comparison between the most important fragrance-shaping carotenoid degradation products in the EOs of the three major types of tobacco—Virginia (flue-cured), Burley (air-cured), and Oriental (sun-cured)—and in the respective traditional aroma products (concrete, resinoid, absolute), all obtained by established technologies and used in the fragrance producing industry. Since the fragrance of tobacco aroma products is directly related to the occurrence of certain classes of volatile compounds, among which are carotenoid-related derivatives, such concise information could have practical importance in the use of tobacco aroma products as natural ingredients in perfumery, cosmetics, and aromatherapy [3].

## 2. Results

The moisture content of the processed plant material was 7.07 ± 0.05% (FCV), 7.23 ± 0.07% (BU), 6.98 ± 0.06% (OR(Kr)), and 6.83 ± 0.04% (OR(Pd7)), respectively. Data about the content of carotenoids (total and β-carotene) in the plant material, as well as the EO yields from the three types of tobacco (mg/g DW), are presented in Table 1. Data reveal differences in carotenoid composition among the tobaccos; the air-cured BU had the highest content of β-carotene (20.34 mg/100 g DW), while the other two tobaccos (flue-cured and sun-cured, both at higher temperatures and shorter periods) had considerably lower β-carotene levels (3.73–12.09 mg/100 g DW). Similar differences were observed in the total carotenoid content among the tobacco types. These results support previous findings about the decisive impact of curing methodology, together with the tobacco-specific enzymatic and microbial processes during curing and fermentation, on carotenoid degradation [13,23,24]. The yields of EO varied significantly, with OR(Kr) tobacco being the highest-yielding plant material (4.44 mg/g DW), followed by OR(Pd7) tobacco (3.01 mg/g DW); this is in line with previous reports and the ranking of Bulgarian oriental tobacco as highly-aromatic [21,22,25].

All EOs were light yellow hydrophobic liquids and had sharp odor. The EO from FCV tobacco had a very intense, balsamic, woody odor with earthy undertones. In contrast, the EO from BU had a mild woody odor with balsamic and floral-like undertones, and the EO from OR had very green odor with slightly smoky and mossy-like and honey-like undertones.

**Figure 2:**
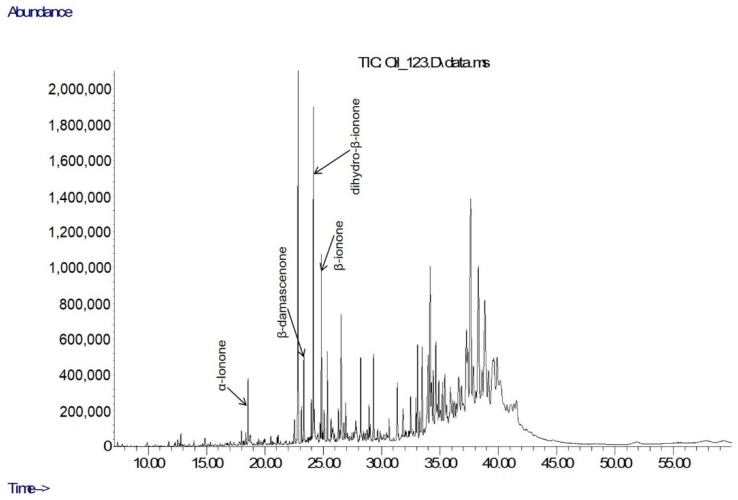
Example of a TIC chromatogram of tobacco EO (OR(Kr)).

The content of the main aroma-impact compounds from β-carotene degradation identified in the EOs [21,22] are presented in Table 2, and an example of the total ion current (TIC) chromatograms (GC-MS) of the analyzed EOs is given on Figure 2. Data in Table 2 are given as relative content (% of TIC) and as calculated true content (mg/100 g DW) in order to provide a better visualization of results, since the latter values are not influenced by co-distilled volatile species or compound characteristics [3]. In the calculations of constituents’ contents response factors were assumed equal for all compounds. The sum of carotenoid derivatives in the EOs (mg/100 g DW) was less than the total carotenoid content in the initial plant material, which suggested that carotenoid transformation during the distillation of EOs involved other products besides the GC-MS quantified volatiles.

Differences were observed among the three types of tobacco, as well as between the two Oriental varieties, corresponding with previously reported differences in the aromatic properties of the tobaccos and their smoke [6,8,13].

## 3. Discussion

As it can be seen in Table 1 and Table 2, there was no proportionate correlation between the initial level of total carotenoids or β-carotene in the leaves and the content of ionones and damascenones in the respective EO. For example, the EO from the air-cured BU tobacco, which had the highest content of total carotenoids and β-carotene, contained ionones and ionone derivatives in concentrations very close to those in the EO from the FCV tobacco, whose leaves had 4–5 times lower initial content of total carotenoids and β-carotene.

Although the identified carotenoid-derived species are not major constituents of the essential oil, their olfactory contribution is decisive for the development of tobacco fragrance [1,5,11,12,20,24,26]. In particular, β-damascenone is described as a powerful fruity-floral odor complex, with apple, plum, rose, raisin, tea, blackcurrant, and tobacco notes [4,24,26]. Two derivatives that differ chemically only by the position of the oxygen function, β-ionone and β-damascone, have completely different olfactory contributions; the odor profile of β-ionone described as cedar-wood, balsamic, violet-raspberry in dilution, while the odor of β-damascone is tobacco, rose, apple, tea, fruity, related to that of β-damascenone [4,24,25,26]. The aroma properties of β-ionone isomer (α-ionone) are described as woody balsamic or violet-raspberry in dilution [4,24,25,26]. In contrast, the related β-ionone alcohol derivative β-ionol has a floral, ambery, and woody scent [4].

The results from this study reveal some differences in damascone derivatives compared with published data on the composition of other tobacco EOs [11,17,18,19,20,27] explainable by plant material origin and processing conditions. For example, Fujimori et al. [11,19] used dichloromethane extraction, steam distillation under reduced pressure and fractionation to obtain the medium-range boiling point fraction (M-fraction) with representative aroma of the EO from good quality Burley tobacco, in which 25 carotenoid-related compounds were identified. In the study by Zhang et al. [28] β-damascenone was 6.15%, 4.72%, and 0.55% and β-ionone was 1.28%, 0.21%, and 0.18%, respectively in the oils and oleoresin obtained by solvent extraction (with petroleum ether) followed by steam distillation, hydrodistillation, and solvent extraction, while α-ionone and dihydro-β-ionone were not identified in neither of products.

The analysis of the available data about the volatile composition of Bulgarian tobaccos revealed that ionone and its derivatives were not identified in the aroma extraction products concrete and resinoid, obtained from the same tobacco varieties as provided by Popova et al. [21,22]. Those aroma products were obtained by extraction of plant materials (1:10, *w/v*) with 95% ethanol at 70 °C (resinoid) and with petroleum ether at 30 °C (concrete), followed by complete removal of the solvents, respectively [21,22] Similarly, ionone and its derivatives were not identified in the commercial-grade absolute from the Bulgarian tobaccos, representing another traditional concentrated tobacco aroma product, obtained from tobacco concrete after extraction with ethanol at low temperature, with the exception of 3-oxo-α-ionol and 3-oxo-7,8-dihydro-α-ionol [3]. Radulović et al. [29] did not identify carotenoid derivatives in the ether and ethyl acetate extracts from oriental and semi-oriental Serbian tobaccos, further stating that they were less complex mixtures than the respective EOs and CO_2_ extracts [30,31,32]. On the other hand, Yokoi and Shimoda [33] found carotenoid degradation products, such as β-damascone, β-damascenone, 3-hydroxy-β-damascone, 3-oxo-α-ionol, and others in low-density polyethylene (LDPE) membrane and direct diethyl ether extracts from Virginia flue-cured tobacco.

It should be stated that various analytical techniques have been applied in the identification of tobacco volatiles, with regard to both plant sample preparation and compound detection, such as solid-phase micro-extraction, accelerated extraction, headspace analysis, simultaneous distillation-extraction, different gas chromatography-mass spectrometry tools, etc., most of which were with much better performance than solvent extraction and hydro- or steam distillation procedures [34,35,36,37,38,39,40,41,42]. All of them contribute substantially to the revealing of the aroma profile of tobacco and provide powerful analytical solutions in contemporary research. Current study and the discussion herein, however, are focused on certain aspects of the composition of established, industrially produced and commercially recognized aroma products from tobacco, in accordance with the classification of Bauer et al. [4], i.e., essential oil, concrete, resinoid, and absolute.

The analysis for carotenoids (total and β-carotene) in the waste plant materials resulting from the hydrodistillation of the EOs in this study (with moisture content adjusted to 7.0 ± 0.5%) detected no quantifiable contents (trace amounts below 0.01 mg/100 g DW), therefore suggesting that the isolation of tobacco EO had been accompanied by near complete degradation of the available carotenoids. On the contrary, the dried waste materials from the extraction of tobacco concrete and resinoid (at the same moisture content) contained carotenoids in amounts comparable to the initial carotenoid values (Table 1). In this way, the comparison between tobacco EO and the tobacco aromatic products obtained by extraction allows assuming that the found differences in the carotenoid-related composition are due to the influence of the processing conditions applied in current and the above-cited studies. Three important aspects of the processing conditions could be outlined as the key driving forces. First, the temperatures during the extraction of concretes (not exceeding 35–40 °С), resinoids (60–70 °С) and absolutes (prepared by further extraction of concrete or resinoid with ethanol and removal of fractions that precipitate at cooling) are considerably lower compared with the temperature of the steam (100 ± 1 °C) during the hydrodistillation of the EOs. Secondly, concretes, resinoids, and absolutes are obtained via extraction with the respective nonpolar (hexane, petroleum ether) or polar (ethanol) solvent without additional pH adjustment, while the studied EOs were distilled from a strongly acidified medium (pH 2), which adds the effect of acidic hydrolysis to the process. There are considerable differences in process duration for the two types of aromatic products, as well, e.g., 1.5 h in total for the extraction of concrete compared to the 3-h hydrodistillation of the EO. These assumptions are supported by previous findings [11,17,20,41] about the effect of thermal degradation, pH of the medium, process duration, and other factors on carotenoid transformations in plant materials.

Despite their intriguing olfactory properties, tobacco EOs are rarely used by the fragrance industry, mostly because of their low yield (typically way below 1% DW). In fact, vectors of the genuine tobacco odor indispensable for fine perfumery are the concentrated extraction products, whose range worldwide is constantly expanding and diversifying, by the introduction of novel extraction techniques or less conventional tobacco materials (types, origins, or quality grades). Therefore, the above observations on the fragrance-shaping carotenoid-derived products in the three Bulgarian tobaccos might be of practical importance in the enhancement of currently applied levels of the influencing technological factors (pH, temperature, etc.,) in aroma production. These results substantiate future research on the transformation of tobacco carotenoids and other aroma-related phytochemicals under optimized extraction conditions facilitating the targeted volatiles’ degradation.

## 4. Materials and Methods

### 4.1. Plant Material

The initial plant materials in this study were leaves from the three types of tobacco (*N. tabacum* L.) produced in Bulgaria: flue-cured Virginia (FCV), Burley (BU), and Oriental (OR) (represented by two varieties “Krumovgrad” (Kr) and “Plovdiv 7” (Pd7)) [21,22]. Gross tobacco samples were obtained directly from the production and curing complexes, from two growing seasons, as follows: FCV—from 3 micro-regions representing North Bulgaria tobacco producing area, BU—from 6 micro-regions representing South Bulgaria tobacco producing area, OR(Kr)—from 8 micro-regions of South Bulgaria Haskovo tobacco producing area, and OR(Pd7)—from the Tobacco and Tobacco Products Institute, in Plovdiv, South Bulgaria. All leaves were handpicked at maturity and cured according to the established technology for the respective tobacco type. The analytical samples represented high quality tobacco material. The moisture of the leaves was determined by drying at 105 °С up to a constant weight [43], and results are presented on a dry weight (DW) basis.

### 4.2. Beta-Carotene Determination

Before analysis, leaves were oven-dried (40 °C; 6 h) and ground in a laboratory mill. Samples of 1.0 g tobacco dust were extracted twice with acetone (30 mL for each extraction) at 25 °С for 30 min. The extracts were diluted in a 50 mL volumetric flask, and carotenoid content was determined spectrophotometrically at wavelengths of 448 and 472 nm [44].

### 4.3. Essential Oil (EO) Isolation

The isolation of tobacco EOs was according to the procedure described previously [21,22]. The EOs were collected by hydrodistillation of 100 g samples for 3 h in a laboratory glass apparatus, according to the British Pharmacopoeia, modified by Balinova and Diakov [45]. The distillation medium was acidified with concentrated sulphuric acid to a pH 2 in order to facilitate the acidic hydrolysis of conjugated aroma and other substances, following the observations of Tsonev and Chenikov [46] that the acid destroyed cell membranes and secretory glands walls, and oil collection was fuller. The EOs obtained were dried over anhydrous sodium sulfate and stored in tightly closed dark vials at 4 °C until analysis. The waste plant materials were removed from the apparatus immediately after completion of EOs hydrodistillation and were dried at 40 °C to a moisture content of 7.0 ± 0.5%. The dried samples were analyzed for carotenoid content according to the method described above, and were used as controls in the carotenoid transformation discussion. The same procedure was applied to the waste plant material resulting from the extraction of concrete and resinoid from the studied tobaccos.

### 4.4. Olfactory Evaluation of The EOs

The olfactory evaluation of the EOs was carried out by three certified perfumers. Each EO (conditioned to room temperature) was dropped on a commercial odor strip and evaluated, then the individual terms for odor description were harmonized [4,25].

### 4.5. Chemical Composition of the EOs

Gas chromatography (GC) analysis was performed on an Agilent 7890A chromatograph (Agilent Technologies Inc., Santa Clara, CA, USA) equipped with an FID detector (Agilent Technologies Inc.), and gas chromatography-mass spectrometry (GC-MS) analysis was on an Agilent 5975C MSD system (Agilent Technologies Inc., Santa Clara, CA, USA). The operational conditions of analysis were as described in [21,22], respectively. The identification of chemical compounds was made by comparison of their mass spectra and retention (Kovat’s) indices with mass spectra library data. The retention indices were estimated using mixtures of homologous series of normal alkanes from C_8_ to C_40_ in hexane. The components identified were arranged according to the retention times and quantities were expressed as a percentage computed using the normalization method of the GC/FID peak areas.

### 4.6. Statistics

All experiments were repeated three times and mean values with their corresponding standard deviation are presented in the tables. Statistical techniques, including ANOVA and Tukey’s multiple comparison test were used to determine the significant differences (*p* < 0.05).

## 5. Conclusions

Based on own experimental results and reference data, a comparative analysis of carotenoids and their derivatives in the most popular tobacco aroma products—EOs, concretes, and resinoids—on the basis of the three major tobacco types grown in Bulgaria was performed. To the best of our knowledge, no such direct comparison has been discussed up to now. Results from the study clearly demonstrate that there are considerable and specific differences between the aroma products. Tobacco EOs contained carotenoid metabolites (α-ionone, β-ionone, β-damascenone, dihydro-β-ionone), and there were significant concentration differences between the three types of tobacco. As anticipated, the EO of the highly aromatic Oriental tobacco was the richest in aroma-related carotenoid derivatives. On the other hand, the solvent extraction of the studied tobacco types for obtaining the traditional aroma products concrete and resinoid, under the well-established processing conditions of industrial production, did not lead to the accumulation of such carotenoid-related compounds in the final products. These results reflect the influence of temperature, pH of the medium, process duration, and probably other factors on the composition of tobacco aroma products. The study provides, in a concise way, a new insight into the composition of EOs and extraction aroma products from the tobaccos produced in Bulgaria, which may be of practical interest to the fragrance industry.

## Figures and Tables

**Table 1:** Chemical indexes of tobacco leaves.

Tobacco	Carotenoids, mg/100 g DW	β-Carotene, mg/100 g DW	Essential Oil, mg/g DW
FCV ^1^	5.93 ± 0.05 ^5,a^	3.73 ± 0.03 ^a^	2.32 ± 0.11 ^a^
BU ^2^	22.23 ± 0.20 ^b^	20.34 ± 0.18 ^b^	2.63 ± 0.12 ^a^
OR(Kr) ^3^	6.27 ± 0.06 ^a^	5.45 ± 0.05 ^c^	4.44 ± 0.14 ^b^
OR(Pd7) ^4^	13.60 ± 0.11 ^c^	12.09 ± 0.10 ^d^	3.01 ± 0.11 ^c^ [22]

^1^ FCV—flue-cured Virginia; ^2^ BU—Burley; ^3^ OR(Kr)—Oriental, variety “Krumovgrad”, ^4^ OR(Pd7)—Oriental, variety “Plovdiv 7”; ^5^ data expressed as mean (*n* = 3) ± standard deviation; ^a–d^ means with different superscripts in a column differed significantly (*p* < 0.05).

**Table 2:** Content of carotenoid derivatives in tobacco EOs.

Tobacco	Component								Ref.
	α-Ionone		β-Ionone		Dihydro-β-Ionone		β-Damascenone		
	% of TIC ^8^	mg/100 g DW	% of TIC	mg/100 g DW	% of TIC	mg/100 g DW	% of TIC	mg/100 g DW	
FCV ^1^	1.37 ± 0.01 ^5,a^	0.61 ± 0.01 ^5,a^	nd ^6^	nd	2.17 ± 0.02 ^a^	0.96 ± 0.02 ^a^	2.92 ± 0.02 ^a^	1.26 ± 0.02 ^a^	[21]
BU ^2^	1.86 ± 0.01 ^b^	0.73 ± 0.01 ^b^	nd	nd	3.10 ± 0.02 ^7, b^	1.19 ± 0.02 ^7,b^	3.53 ± 0.02 ^b^	1.35 ± 0.02 ^b^	[21]
OR(Kr) ^3^	0.91 ± 0.01 ^c^	0.20 ± 0.00 ^c^	2.76 ± 0.02	1.08 ± 0.02	5.92 ± 0.03 ^c^	1.34 ± 0.03 ^c^	1.59 ± 0.01 ^c^	0.36 ± 0.01 ^c^	[21]
OR(Pd7) ^4^	4.32 ± 0.02 ^d^	1.43 ± 0.02 ^d^	nd	nd	5.19 ± 0.03 ^c^	1.73 ± 0.03 ^d^	3.71 ± 0.02 ^b^	1.23 ± 0.02 ^a^	[22]

^1^ FCV—flue-cured Virginia; ^2^ BU—Burley; ^3^ OR(Kr)—Oriental, variety “Krumovgrad”; ^4^ OR(Pd7)—Oriental, variety “Plovdiv 7”; ^5^ data expressed as mean (*n* = 3) ± standard deviation; ^6^ nd—not determined; ^7^ sum of two isomers; ^8^ identified at >0.05% of TIC; ^a–d^ means with different superscripts in a column differed significantly (*p* < 0.05).
